# 2-{2-[2-(1,3-Dioxoisoindol-2-yl)eth­oxy]eth­yl}isoindole-1,3-dione

**DOI:** 10.1107/S1600536811018496

**Published:** 2011-05-20

**Authors:** Samat Talipov, Abdurasul Yuldashev, Zakirjon Karimov, Kambarali Turgunov, Bakhtiyar Ibragimov

**Affiliations:** a Institute of Bioorganic Chemistry, Academy of Sciences of Uzbekistan, Mirzo Ulugbek Str. 83, Tashkent 100125, Uzbekistan; b The National University of Uzbekistan named after Mirzo Ulugbek, Faculty of Chemistry, University Str. 6, Tashkent 100779, Uzbekistan; c Tashkent Institute of Irrigation and Melioration, Qori-Niyoziy Str. 39, Tashkent 100000, Uzbekistan; d S. Yunusov Institute of the Chemistry of Plant Substances, Academy of Sciences of Uzbekistan, Mirzo Ulugbek Str. 77, Tashkent 100170, Uzbekistan

## Abstract

In the mol­ecule of the title compound, C_20_H_16_N_2_O_5_, the phthalimide fragments are almost planar, with r.m.s. deviations of 0.018 and 0.020 Å, and make a dihedral angle of 53.64 (3)°. The mol­ecular and crystal structures are stabilized by a weak inter­molecular C—H⋯O, C—H⋯π and C=O⋯π [2.883 (1) Å] inter­actions and aromatic π–π stacking inter­actions with a centroid–centroid distance of 3.6189 (7) Å.

## Related literature

For related structures, see: Valle *et al.* (1986 ▶); Sheng *et al.* (2007 ▶). For the preparation, see: Yatsimirskii *et al.* (1987 ▶).
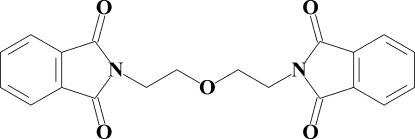


## Experimental

### 

#### Crystal data

C_20_H_16_N_2_O_5_
*M* *_r_* = 364.35
Monoclinic, 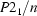

*a* = 10.8928 (1) Å
*b* = 11.9656 (1) Å
*c* = 14.3572 (2) Å
β = 111.633 (1)°
*V* = 1739.49 (3) Å^3^
*Z* = 4
Cu *K*α radiation
μ = 0.85 mm^−1^
*T* = 293 K
0.40 × 0.30 × 0.20 mm

#### Data collection

Oxford Diffraction Xcalibur Ruby diffractometer
Absorption correction: multi-scan (*CrysAlis PRO*; Oxford Diffraction, 2009 ▶) *T*_min_ = 0.333, *T*_max_ = 1.000
14673 measured reflections
3575 independent reflections
3075 reflections with *I* > 2σ(*I*)
*R*_int_ = 0.025

#### Refinement

*R*[*F* ^2^ > 2σ(*F* ^2^)] = 0.038
*wR*(*F* ^2^) = 0.115
*S* = 1.07
3575 reflections
245 parameters
H-atom parameters constrained
Δρ_max_ = 0.21 e Å^−3^
Δρ_min_ = −0.14 e Å^−3^

### 

Data collection: *CrysAlis PRO* (Oxford Diffraction, 2009 ▶); cell refinement: *CrysAlis PRO*; data reduction: *CrysAlis PRO*; program(s) used to solve structure: *SHELXS97* (Sheldrick, 2008 ▶); program(s) used to refine structure: *SHELXL97* (Sheldrick, 2008 ▶); molecular graphics: *XP* in *SHELXTL* (Sheldrick, 2008 ▶); software used to prepare material for publication: *SHELXL97*.

## Supplementary Material

Crystal structure: contains datablocks I, global. DOI: 10.1107/S1600536811018496/gw2102sup1.cif

Structure factors: contains datablocks I. DOI: 10.1107/S1600536811018496/gw2102Isup2.hkl

Supplementary material file. DOI: 10.1107/S1600536811018496/gw2102Isup3.cml

Additional supplementary materials:  crystallographic information; 3D view; checkCIF report

## Figures and Tables

**Table 1:** Hydrogen-bond geometry (Å, °) *Cg*3 and *Cg*4 are the centroids of the C2/C3/C5–C8 and C14/C15/C17–C20 rings, respectively.

*D*—H⋯*A*	*D*—H	H⋯*A*	*D*⋯*A*	*D*—H⋯*A*
C5—H5*A*⋯O5^i^	0.93	2.47	3.171 (2)	132
C19—H19*A*⋯O5^ii^	0.93	2.45	3.286 (4)	150
C11—H11*B*⋯*Cg*3^iii^	0.97	2.84	3.624 (2)	139
C12—H12*B*⋯*Cg*4^iv^	0.97	2.94	3.567 (2)	123

Symmetry codes: (i) 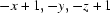; (ii) 
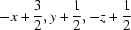
; (iii) 
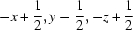
; (iv) 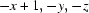.

**Fig. 1.**
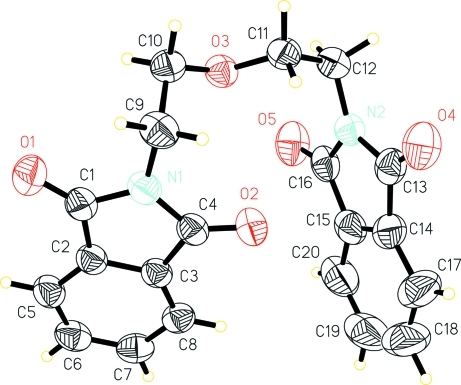
Molecular structure of the title compound with 50% probability displacement ellipsoids for non-H atoms.

**Fig. 2.**
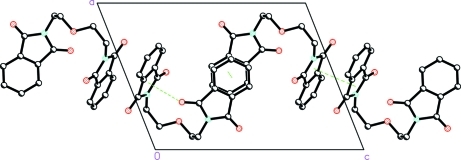
View of the crystal structure along the b-axis showing a C=O···π and π···π stacking interactions (dashed lines).

## supplementary crystallographic information

### Comment

The asymmetric unit contains one molecule of the title compound (Figure 1). In the molecule phthalimide fragments are planar, with r.m.s. deviations of 0.018Å and 0.020Å, respectively. The angle between planes is 53.64 (3)°. The observed structure is stabilized by weak C—H···O and C-H···π(ring) hydrogen bonds (Table 1), as well as C=O···π(ring) (C4=O2···Cg2 distance is 2.883 (1), where Cg2 is N2C13C14C15C16 ring centroid) and aromatic π···π stacking interactions. A centrosymmetric π···π stacking interactions are observed between maleimide rings (Cg2···Cg2^i^ distance is 3.4805 (9) Å, where i = *1-x, -y, -z*) and benzene rings (Cg3···Cg3^ii^ distance 3.6189 (7)Å, where Cg3 is C2C3C5C6C7C8 ring centroid, ii = *1-x, -y, 1-z*) (Figure 2).

### Experimental

The title compound is received by the slightly modified technique (Yatsimirskii *et al.*,1987). 24 g (0.12 mole) potassium phthalimide and 8 ml (0.05 mole) β,β'-dichloroethyl ether were taken in a three-necked round-battomed flask supplied with a reflux condenser and a mechanical stirrer. Reaction is carried out at 463-473 K within 2.5 hours by stirring. After corresponding chemical treatments (Yatsimirskii *et al.*,1987) reaction product was recrystallized from 1:1 mixture of ethanol and chloroform. 13.99 g (56 %) title compound , with m.p. of 421-423 K was received.

### Refinement

Carbon-bound H atoms were positioned geometrically and treated as riding on their C atoms, with C—H distances of 0.93 Å (aromatic) and 0.97 Å (CH_2_) and were refined with Uiso(H)=1.2Ueq(C).

### Crystal data

**Table:**

C_20_H_16_N_2_O_5_	*F*(000) = 760
*M**_r_* = 364.35	*D*_x_ = 1.391 Mg m^−^^3^
Monoclinic, *P*2_1_/*n*	Cu *K*α radiation, λ = 1.54180 Å
Hall symbol: -P 2yn	Cell parameters from 9302 reflections
*a* = 10.8928 (1) Å	θ = 3.3–75.5°
*b* = 11.9656 (1) Å	µ = 0.85 mm^−^^1^
*c* = 14.3572 (2) Å	*T* = 293 K
β = 111.633 (1)°	Prism, colourless
*V* = 1739.49 (3) Å^3^	0.40 × 0.30 × 0.20 mm
*Z* = 4	

### Data collection

**Table:**

Oxford Diffraction Xcalibur Ruby diffractometer	3575 independent reflections
Radiation source: Enhance (Cu) X-ray Source	3075 reflections with *I* > 2σ(*I*)
graphite	*R*_int_ = 0.025
Detector resolution: 10.2576 pixels mm^-1^	θ_max_ = 75.6°, θ_min_ = 4.4°
ω scans	*h* = −13→10
Absorption correction: multi-scan (*CrysAlis PRO*; Oxford Diffraction, 2009)	*k* = −14→15
*T*_min_ = 0.333, *T*_max_ = 1.000	*l* = −14→17
14673 measured reflections	

### Refinement

**Table:**

Refinement on *F*^2^	Secondary atom site location: difference Fourier map
Least-squares matrix: full	Hydrogen site location: inferred from neighbouring sites
*R*[*F*^2^ > 2σ(*F*^2^)] = 0.038	H-atom parameters constrained
*wR*(*F*^2^) = 0.115	*w* = 1/[σ^2^(*F*_o_^2^) + (0.0682*P*)^2^ + 0.1804*P*] where *P* = (*F*_o_^2^ + 2*F*_c_^2^)/3
*S* = 1.07	(Δ/σ)_max_ < 0.001
3575 reflections	Δρ_max_ = 0.21 e Å^−^^3^
245 parameters	Δρ_min_ = −0.14 e Å^−^^3^
0 restraints	Extinction correction: *SHELXL*, Fc^*^=kFc[1+0.001xFc^2^λ^3^/sin(2θ)]^-1/4^
Primary atom site location: structure-invariant direct methods	Extinction coefficient: 0.0094 (6)

### Special details

**Table:**

Geometry. All esds (except the esd in the dihedral angle between two l.s. planes) are estimated using the full covariance matrix. The cell esds are taken into account individually in the estimation of esds in distances, angles and torsion angles; correlations between esds in cell parameters are only used when they are defined by crystal symmetry. An approximate (isotropic) treatment of cell esds is used for estimating esds involving l.s. planes.
Refinement. Refinement of F^2^ against ALL reflections. The weighted R-factor wR and goodness of fit S are based on F^2^, conventional R-factors R are based on F, with F set to zero for negative F^2^. The threshold expression of F^2^ > 2sigma(F^2^) is used only for calculating R-factors(gt) etc. and is not relevant to the choice of reflections for refinement. R-factors based on F^2^ are statistically about twice as large as those based on F, and R- factors based on ALL data will be even larger.

### Fractional atomic coordinates and isotropic or equivalent isotropic displacement parameters (Å^2^)

**Table:**

	*x*	*y*	*z*	*U*_iso_*/*U*_eq_	
N1	0.20673 (10)	0.08438 (9)	0.31107 (8)	0.0482 (3)	
N2	0.37536 (10)	−0.01311 (9)	0.06449 (8)	0.0456 (3)	
O1	0.14477 (11)	0.03279 (11)	0.44214 (8)	0.0705 (3)	
O2	0.32443 (10)	0.15162 (9)	0.21829 (7)	0.0564 (3)	
O3	0.19329 (8)	−0.09719 (7)	0.16698 (6)	0.0470 (2)	
O4	0.26607 (11)	0.11689 (10)	−0.05545 (9)	0.0714 (3)	
O5	0.52801 (11)	−0.10263 (11)	0.19907 (8)	0.0726 (3)	
C1	0.22559 (13)	0.07069 (11)	0.41171 (10)	0.0491 (3)	
C2	0.36170 (13)	0.11145 (10)	0.46882 (9)	0.0445 (3)	
C3	0.41737 (12)	0.14479 (10)	0.40088 (9)	0.0423 (3)	
C4	0.31715 (12)	0.12978 (10)	0.29806 (9)	0.0444 (3)	
C5	0.43090 (15)	0.11709 (11)	0.57083 (10)	0.0524 (3)	
H5A	0.3929	0.0948	0.6162	0.063*	
C6	0.55940 (14)	0.15730 (12)	0.60309 (10)	0.0554 (3)	
H6A	0.6084	0.1625	0.6714	0.067*	
C7	0.61590 (14)	0.18991 (11)	0.53518 (11)	0.0541 (3)	
H7A	0.7024	0.2161	0.5588	0.065*	
C8	0.54558 (13)	0.18413 (10)	0.43237 (10)	0.0485 (3)	
H8A	0.5833	0.2059	0.3867	0.058*	
C9	0.08711 (13)	0.05067 (13)	0.22883 (11)	0.0547 (3)	
H9A	0.0767	0.0963	0.1706	0.066*	
H9B	0.0114	0.0638	0.2476	0.066*	
C10	0.09011 (13)	−0.07152 (12)	0.20170 (10)	0.0514 (3)	
H10A	0.1008	−0.1169	0.2601	0.062*	
H10B	0.0061	−0.0910	0.1500	0.062*	
C11	0.15869 (12)	−0.07463 (12)	0.06348 (9)	0.0483 (3)	
H11A	0.1335	0.0032	0.0500	0.058*	
H11B	0.0839	−0.1204	0.0246	0.058*	
C12	0.27467 (14)	−0.09957 (12)	0.03325 (10)	0.0521 (3)	
H12A	0.3137	−0.1702	0.0627	0.062*	
H12B	0.2435	−0.1074	−0.0390	0.062*	
C13	0.35959 (14)	0.09081 (11)	0.01785 (10)	0.0511 (3)	
C14	0.47835 (15)	0.15636 (13)	0.07673 (12)	0.0603 (4)	
C15	0.55826 (14)	0.08926 (15)	0.15356 (11)	0.0613 (4)	
C16	0.49218 (13)	−0.02092 (13)	0.14666 (10)	0.0514 (3)	
C17	0.5109 (2)	0.26603 (16)	0.06645 (19)	0.0904 (7)	
H17A	0.4569	0.3112	0.0149	0.108*	
C18	0.6273 (3)	0.3050 (2)	0.1364 (3)	0.1217 (11)	
H18A	0.6528	0.3782	0.1312	0.146*	
C19	0.7070 (3)	0.2397 (3)	0.2133 (2)	0.1250 (12)	
H19A	0.7838	0.2700	0.2597	0.150*	
C20	0.67502 (18)	0.1286 (2)	0.22309 (15)	0.0915 (7)	
H20A	0.7298	0.0833	0.2741	0.110*	

### Atomic displacement parameters (Å^2^)

**Table:**

	*U*^11^	*U*^22^	*U*^33^	*U*^12^	*U*^13^	*U*^23^
N1	0.0457 (5)	0.0535 (6)	0.0441 (6)	−0.0019 (4)	0.0151 (4)	−0.0071 (5)
N2	0.0453 (5)	0.0485 (6)	0.0437 (5)	0.0008 (4)	0.0174 (4)	−0.0006 (4)
O1	0.0660 (7)	0.0886 (8)	0.0673 (7)	−0.0175 (6)	0.0368 (6)	−0.0077 (6)
O2	0.0646 (6)	0.0640 (6)	0.0431 (5)	−0.0003 (5)	0.0229 (4)	−0.0004 (4)
O3	0.0425 (4)	0.0523 (5)	0.0423 (5)	−0.0002 (4)	0.0109 (4)	−0.0008 (4)
O4	0.0706 (7)	0.0761 (7)	0.0661 (7)	0.0161 (6)	0.0235 (6)	0.0212 (6)
O5	0.0626 (6)	0.0927 (8)	0.0602 (6)	0.0190 (6)	0.0200 (5)	0.0223 (6)
C1	0.0514 (7)	0.0497 (7)	0.0498 (7)	−0.0015 (5)	0.0230 (6)	−0.0062 (5)
C2	0.0505 (6)	0.0402 (6)	0.0429 (6)	0.0016 (5)	0.0175 (5)	−0.0023 (5)
C3	0.0477 (6)	0.0364 (5)	0.0423 (6)	0.0028 (5)	0.0161 (5)	−0.0017 (4)
C4	0.0481 (6)	0.0423 (6)	0.0439 (6)	0.0032 (5)	0.0183 (5)	−0.0038 (5)
C5	0.0642 (8)	0.0498 (7)	0.0421 (7)	0.0009 (6)	0.0183 (6)	0.0007 (5)
C6	0.0626 (8)	0.0480 (7)	0.0431 (7)	0.0028 (6)	0.0046 (6)	−0.0008 (5)
C7	0.0482 (7)	0.0448 (7)	0.0592 (8)	−0.0001 (5)	0.0080 (6)	−0.0011 (6)
C8	0.0492 (6)	0.0418 (6)	0.0551 (7)	0.0002 (5)	0.0200 (6)	−0.0002 (5)
C9	0.0416 (6)	0.0640 (8)	0.0531 (7)	0.0026 (6)	0.0111 (6)	−0.0078 (6)
C10	0.0432 (6)	0.0584 (8)	0.0508 (7)	−0.0081 (5)	0.0155 (5)	−0.0058 (6)
C11	0.0424 (6)	0.0540 (7)	0.0423 (6)	−0.0051 (5)	0.0085 (5)	−0.0002 (5)
C12	0.0562 (7)	0.0503 (7)	0.0493 (7)	−0.0044 (6)	0.0189 (6)	−0.0074 (5)
C13	0.0561 (7)	0.0511 (7)	0.0542 (7)	0.0052 (6)	0.0299 (6)	0.0029 (6)
C14	0.0673 (9)	0.0585 (8)	0.0724 (9)	−0.0078 (7)	0.0461 (8)	−0.0103 (7)
C15	0.0520 (7)	0.0842 (10)	0.0571 (8)	−0.0144 (7)	0.0312 (7)	−0.0201 (7)
C16	0.0451 (6)	0.0700 (9)	0.0421 (6)	0.0044 (6)	0.0194 (5)	−0.0004 (6)
C17	0.1166 (16)	0.0630 (10)	0.1294 (17)	−0.0240 (10)	0.0896 (15)	−0.0194 (10)
C18	0.152 (3)	0.0986 (18)	0.165 (3)	−0.0711 (18)	0.118 (2)	−0.0610 (18)
C19	0.1109 (19)	0.170 (3)	0.124 (2)	−0.091 (2)	0.0779 (17)	−0.086 (2)
C20	0.0641 (10)	0.144 (2)	0.0739 (11)	−0.0364 (11)	0.0341 (9)	−0.0386 (12)

### Geometric parameters (Å, °)

**Table:**

N1—C1	1.3922 (16)	C8—H8A	0.9300
N1—C4	1.3937 (17)	C9—C10	1.517 (2)
N1—C9	1.4561 (16)	C9—H9A	0.9700
N2—C16	1.3830 (16)	C9—H9B	0.9700
N2—C13	1.3926 (17)	C10—H10A	0.9700
N2—C12	1.4531 (17)	C10—H10B	0.9700
O1—C1	1.2062 (16)	C11—C12	1.5092 (19)
O2—C4	1.2055 (15)	C11—H11A	0.9700
O3—C11	1.4178 (15)	C11—H11B	0.9700
O3—C10	1.4209 (16)	C12—H12A	0.9700
O4—C13	1.2051 (17)	C12—H12B	0.9700
O5—C16	1.2075 (18)	C13—C14	1.482 (2)
C1—C2	1.4873 (18)	C14—C17	1.381 (2)
C2—C5	1.3798 (18)	C14—C15	1.382 (2)
C2—C3	1.3836 (17)	C15—C20	1.378 (2)
C3—C8	1.3823 (18)	C15—C16	1.488 (2)
C3—C4	1.4884 (17)	C17—C18	1.376 (4)
C5—C6	1.388 (2)	C17—H17A	0.9300
C5—H5A	0.9300	C18—C19	1.369 (4)
C6—C7	1.387 (2)	C18—H18A	0.9300
C6—H6A	0.9300	C19—C20	1.395 (4)
C7—C8	1.3917 (19)	C19—H19A	0.9300
C7—H7A	0.9300	C20—H20A	0.9300
			
C1—N1—C4	112.37 (10)	O3—C10—H10B	108.9
C1—N1—C9	123.68 (11)	C9—C10—H10B	108.9
C4—N1—C9	123.90 (11)	H10A—C10—H10B	107.8
C16—N2—C13	112.47 (11)	O3—C11—C12	109.66 (10)
C16—N2—C12	124.58 (11)	O3—C11—H11A	109.7
C13—N2—C12	122.82 (11)	C12—C11—H11A	109.7
C11—O3—C10	112.80 (10)	O3—C11—H11B	109.7
O1—C1—N1	124.89 (13)	C12—C11—H11B	109.7
O1—C1—C2	129.50 (13)	H11A—C11—H11B	108.2
N1—C1—C2	105.61 (11)	N2—C12—C11	112.77 (11)
C5—C2—C3	121.62 (12)	N2—C12—H12A	109.0
C5—C2—C1	130.14 (12)	C11—C12—H12A	109.0
C3—C2—C1	108.24 (11)	N2—C12—H12B	109.0
C8—C3—C2	121.35 (12)	C11—C12—H12B	109.0
C8—C3—C4	130.44 (12)	H12A—C12—H12B	107.8
C2—C3—C4	108.21 (11)	O4—C13—N2	124.47 (13)
O2—C4—N1	125.04 (12)	O4—C13—C14	129.80 (14)
O2—C4—C3	129.43 (12)	N2—C13—C14	105.74 (12)
N1—C4—C3	105.53 (10)	C17—C14—C15	121.74 (18)
C2—C5—C6	117.40 (13)	C17—C14—C13	130.25 (18)
C2—C5—H5A	121.3	C15—C14—C13	107.95 (13)
C6—C5—H5A	121.3	C20—C15—C14	121.39 (18)
C7—C6—C5	121.13 (12)	C20—C15—C16	130.21 (18)
C7—C6—H6A	119.4	C14—C15—C16	108.34 (13)
C5—C6—H6A	119.4	O5—C16—N2	124.80 (14)
C6—C7—C8	121.21 (13)	O5—C16—C15	129.70 (14)
C6—C7—H7A	119.4	N2—C16—C15	105.51 (12)
C8—C7—H7A	119.4	C18—C17—C14	116.6 (2)
C3—C8—C7	117.29 (12)	C18—C17—H17A	121.7
C3—C8—H8A	121.4	C14—C17—H17A	121.7
C7—C8—H8A	121.4	C19—C18—C17	122.3 (2)
N1—C9—C10	112.19 (11)	C19—C18—H18A	118.8
N1—C9—H9A	109.2	C17—C18—H18A	118.8
C10—C9—H9A	109.2	C18—C19—C20	121.2 (2)
N1—C9—H9B	109.2	C18—C19—H19A	119.4
C10—C9—H9B	109.2	C20—C19—H19A	119.4
H9A—C9—H9B	107.9	C15—C20—C19	116.8 (2)
O3—C10—C9	113.15 (11)	C15—C20—H20A	121.6
O3—C10—H10A	108.9	C19—C20—H20A	121.6
C9—C10—H10A	108.9		

### Hydrogen-bond geometry (Å, °)

**Table:**

Cg3 and Cg4 are the centroids of the C2/C3/C5–C8 and C14/C15/C17–C20 rings, respectively.

**Table:**

*D*—H···*A*	*D*—H	H···*A*	*D*···*A*	*D*—H···*A*
C5—H5A···O5^i^	0.93	2.47	3.171 (2)	132
C19—H19A···O5^ii^	0.93	2.45	3.286 (4)	150
C11—H11B···Cg3^iii^	0.97	2.84	3.624 (2)	139
C12—H12B···Cg4^iv^	0.97	2.94	3.567 (2)	123

Symmetry codes: (i) −*x*+1, −*y*, −*z*+1; (ii) −*x*+3/2, *y*+1/2, −*z*+1/2; (iii) −*x*+1/2, *y*−1/2, −*z*+1/2; (iv) −*x*+1, −*y*, −*z*.
